# Halophyte *Artemisia caerulescens* L.: Metabolites from In Vitro Shoots and Wild Plants

**DOI:** 10.3390/plants11081081

**Published:** 2022-04-15

**Authors:** Ylenia Pieracci, Martina Vento, Luisa Pistelli, Tiziana Lombardi, Laura Pistelli

**Affiliations:** 1 Dipartimento di Farmacia, Università di Pisa, Via Bonanno 6, 56126 Pisa, Italy; ylenia.pieracci@phd.unipi.it (Y.P.); m.vento1@studenti.unipi.it (M.V.); luisa.pistelli@unipi.it (L.P.); 2 Centro Interdipartimentale di Ricerca Nutraceutica e Alimentazione per la Salute ‘NUTRAFOOD’, Università di Pisa, Via del Borghetto 80, 56124 Pisa, Italy; 3 Dipartimento di Scienze Agrarie, Alimentari e Agro-Alimentari, Università di Pisa, Via del Borghetto 80, 56124 Pisa, Italy; tiziana.lombardi@unipi.it; 4 Centre for Climate Change Impact (CIRSEC), University of Pisa, Via del Borghetto 80, 56124 Pisa, Italy

**Keywords:** shoot proliferation, polyphenols, antioxidant activity, essential oils, HS-SPME, GC-MS, PCA, HCA

## Abstract

Halophyte plants are potential resources to deal with the increasing soil salinity determined by climatic change. In this context, the present study aimed to investigate the germplasm conservation of *Artemisia caerulescens* collected in the San Rossore Estate (Pisa, Italy) through in vitro culture, biochemical properties, and the phytochemical composition of the volatile fraction of both in vitro shoots and different organs of wild plants (leaves, young and ripe inflorescences). The best medium tested for the shoot proliferation was MS, with the addition of 1 μM BA. Total polyphenol content and antioxidant activity were noticeable in both the inflorescences, while leaves and in vitro shoots showed lower amounts. Concerning the phytochemical investigation, the headspaces (HSs) and the essential oils (EOs) were characterized by oxygenated monoterpenes as the main chemical class of compounds in all samples, and with α- and β-thujone as the major constituents. However, the EOs were characterized by noticeable percentages of phenylpropanoids (23.6–28.8%), with brevifolin as the unique compound, which was not detected in the spontaneous volatile emissions of the same parts of the wild plant. Good amounts of EOs were obtained from different organs of the wild plant, comprising between 0.17% and 0.41% of the young and ripe inflorescences, respectively.

## 1. Introduction

*Artemisia caerulescens* L. is a perennial aromatic shrub that belongs to the genus *Artemisia* L. and is included in the Asteraceae family [1]. 

The genus *Artemisia* L. comprises more than 500 perennial herb species, and it is widespread in the Northern hemisphere, mainly in the arid and semiarid areas of Asia, Europe, and North America [2,3]. Most of the species belonging to the *Artemisia* genus are medicinal or aromatic herbs or shrubs, characterized by a pungent smell and a bitter taste due to the presence of terpenoids, typical components of their essential oils (EOs) [4]. This genus has achieved increasing phytochemical attention for its wide range of biological activities, attributable to the presence of several classes of active compounds such as terpenes found in essential oil, phenolic compounds, flavonoids, and sesquiterpene lactones [3,5]. The *Artemisia* species have been employed since ancient times for the treatment of different disorders, including malaria, hepatitis, cancer, inflammation as well as antibiotics [2], antiparasitic, insecticidal, anti-asthmatic, and antiepileptic remedies, among others [5]. Essential oils (EOs), besides the non-volatile compounds, are important secondary metabolites obtained from several plants belonging to this genus [4]. They are complex mixtures of volatile compounds widely used in traditional and modern medicine; likewise, they are used for cosmetic and pharmaceutical applications [2]. In recent times, EOs have gained increasing interest due to consumers’ preference for natural products [6] and have been used in many fields of applications, from human health to agriculture, thanks to their broad spectrum of bioactivities, which is attributable to their chemical composition [7,8,9,10]. 

**Figure 1:**
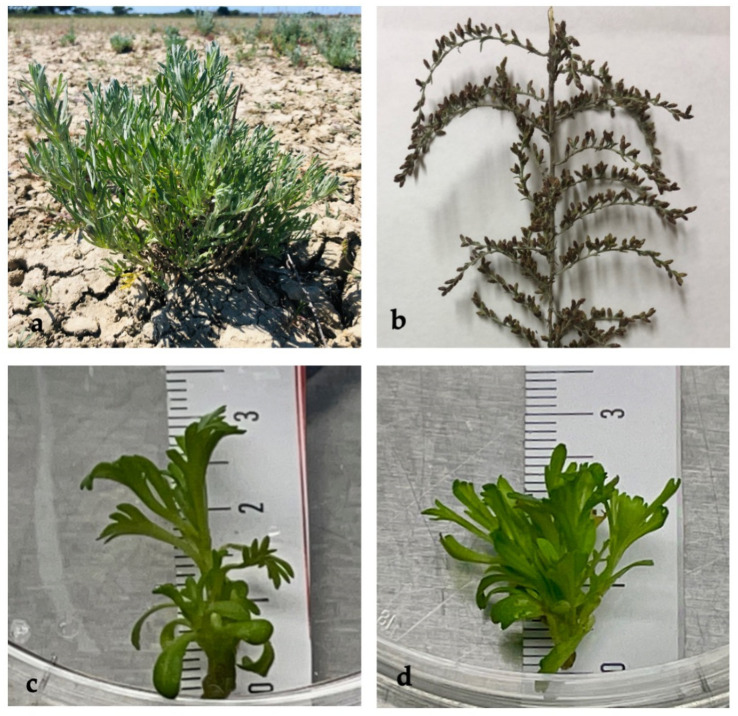
(**a**) *Artemisia caerulescens* in its natural habitat in the San Rossore estate (photo by Laura Pistelli); (**b**) ripe inflorescence (**c**) 4-week-old shoots in MSO medium; (**d**) 4-week-old shoots in 4 μM MS-BA medium.

The genus *Artemisia* comprises a wide range of species that have been subjected to phytochemical attention for their chemical diversity and EO production. These differences are attributable to their ability to colonize areas with different kinds of vegetation and ecological conditions, thus leading to different morphological and biological features [11,12]. Among the countless species of *Artemisia*, *A. caerulescens* is a wild aromatic species of great environmental interest due to its ability to grow and reproduce in selected areas of the central and western Mediterranean, which is characterized by highly saline soils [13]. This is a perennial, bushy, more or less tomentose plant, with a strong aromatic odour, very branched erect stems, and bearing both whole and 1–2-pinnate leaves. The flower heads, all with tubular flowers, are not very showy and arranged in a dense panicle (Figure 1a,b). The fruits are oval achenes, smooth and without pappus. The species is in fact considered a true halophyte [14] and is listed as a characterizing and/or diagnostic species of saline EU Habitats [15,16]. Despite species showed a remarkable interest in the environmental and conservation fields, even with evidence of some particular aspects in its behaviour such as the interaction between propagation by seed and seasonal fluctuations of salinity [17], to date, there were very few studies in literature. This information deficit affects various fields of study, including those related to its phytochemical profile and medicinal properties, which are the subject of the present research.

As a part of an ongoing project, which deals with the cultivation and analysis of halophyte species growing in the salt marshes on the coast of Tuscany, the present study aims to investigate preliminary evidence from in vitro cultures of *A. caerulescens* collected in the San Rossore Estate (Pisa, Italy) in order to verify the possibility of its germplasm conservation, thus avoiding environmental depletion and allowing for the selection of the most promising cultivation lines. Moreover, biochemical and phytochemical analyses were performed on both the in vitro plant culture and different parts of the wild plant, particularly the leaves, young flowering tops as well as ripe ones.

## 2. Results and Discussion

### 2.1. In Vitro Shoot Proliferation

*A. caerulescens* grows spontaneously in the San Rossore Estate, in salty soil close to the sea. Few studies are available on its ecological adaptability or on its growth strategy [8,12]. Aerial parts were harvested in June and September 2021, at the vegetative and flowering stages, and then used for in vitro shoot proliferation to avoid the depletion of the wild plants for future analysis. The same “mother” plants were also used for the biochemical and phytochemical analyses.

The sterilization step was repeated for each single mother plant; the obtained results are summarized in Table 1. The initial contamination ranged between 10% and 90% of the explants and appeared a few weeks after the sterilization process. The high contamination could be due to the natural and protected habitat of the spontaneous plants and the lack of a pre-treatment with pesticides before the in vitro cultivation, as reported in some literature [18]. The shoots proliferated in either Murashige Skoog basal medium [19] (MS0) or in MS with the addition of cytokinin (MS-BA). Shoot induction was performed with the addition of cytokinin 6-benzyl-aminopurine (BA) as a unique growth regulator, which was a pivotal experiment investigating the ability of A. *caerulescens* in shoot proliferation.

After 4 weeks of cultivation, the MS0 explants showed a lower proliferation (one shoot/explant), while the explants grown in the presence of BA showed better proliferation, with 3–5 shoots in independent experiments. However, the addition of BA produced shorter shoots (1.2–1.5 cm in length) than those of MS0 (2.67–3.5 cm) (Figure 1c,d, respectively).

Therefore, other shoots were cultured with MS, combined with different concentrations of BA (1, 2, or 4 μM). The highest proliferation of shoots was obtained with the lowest BA concentration (8.1 shoots per explant), followed by the concentration of 2 μM and 4 μM BA. The highest shoot length was achieved with the use of the MS0 medium (range, 2.67–3.5 cm), and the shortest shoots (0.76 cm) with the 4 μM BA. The effect of the addition of cytokinin is well-known to increase the number of shoots in other species. In *A. vulgaris*, the shoots proliferated in liquid MS with 0.44–8.88 µM BA, and the best effect on shoot multiplication was obtained with the addition of 4.44 µM BA, producing 85.5 shoots/explant at 500 mL flask capacity and with an average length of 12.2 cm [20]. The extraordinary data differed from our results due to the methodology and the species used. Usually, the solid medium is used for shoot induction and proliferation. In *A. granatensis,* the highest axillary shoot proliferation rate was achieved with a solid medium and the addition of 0.44 μM BA, although the shoots exhibited short lengths (0.5 cm). On the contrary, the longest length of the main shoots (2.1 cm) was achieved with MSA0 [21], which did not differ from our results on shoot induction (MS0, 2.67–3.5 cm). 

However, a wide range of literature on other *Artemisia* species has highlighted the use of both cytokinins (BA) and other growth regulators in producing high shoot proliferation. In *A. annua*, BA (0.5–1.0 mg/L, e.g., 2–4 μM) stimulated the highest shoot proliferation, although other types of hormones (e.g., Gibberellin acid, GA_3_) also induced the development of shoots from the tip explants [22]. *A. absinthium* nodal explants cultivated in the presence of 0.5 mg/L BA with 0.25 mg/L Kinetin (kn) produced shoots with a length (6.0 ± 0.52 cm) of 3.25 ± 0.42 cm [23], although the unique addition of BA produced the highest shoot number (4.5) when the concentration was 0.5 mg/L (e.g., 2 μM) [23]. In other reports, the proliferation of shoots has been correlated with the production of secondary metabolites. In *A. alba,* the simultaneous presence of indole-3-butyric acid (IBA) and BA in the medium promoted the development of shoots and roots and phenolic compounds [24]. Low BA concentration in combination with different IBA concentrations increased the amount of phenolic compounds as compared with the basal medium without plant growth regulators [24,25]. 

The different responses of the *Artemisia* species to the various culture media indicate that the pivotal results obtained in *A. caerulescens* with the unique addition of BA can be improved by the further addition of growth regulators. 

No data about the rooting are reported in this manuscript since the pivotal experiments were performed to obtain higher shoot proliferation and thus to investigate the production of metabolites by in vitro shoots. Rooting experiments will be performed in future experiments and will be explored combination with various growth regulators to improve the propagation technique.

### 2.2. Biochemical Analysis 

The *A. caerulescens* wild plants were collected during the summer period to obtain different aerial organs (leaves, and young and ripe inflorescences). The photosynthetic pigments Chlorophyll a and b (chl a 930.03 and chl b 182.27 µg g^−1^ FW, respectively; Table 2) were revealed in amounts that have already been observed for other *Artemisia* sp. such as *A*. *pauciflora*, *A*. *lerchiana*, and *A*. *santonica*, although they were often referred to in dry weight [12,26]. In fact, an estimation of the total chlorophyll content of *A. caerulescens* based on dry material (4.69 mg g^−1^ dry weight) is similar to that of *A. santonica* (4.8 mg g^−1^ dry weight) [12]. Inflorescences contained different pigments depending on the flowering stage: the blossoms showed higher chlorophyll content than the ripe ones (376.99 and 231.31 µg g^−1^ FW, respectively). The photosynthetic pigments were evaluated in other *Artemisia* sp. tolerant to salinity; the *A. absinthium* young plants grown in salty conditions showed similar values to those of *A. caerulescens* [27]. 

Other pigments, carotenoids, and anthocyanins were detected and related to antioxidant activity. Leaves of wild plants showed the highest amount of carotenoids (294.09 µg g^−1^ FW), probably associated with the photoprotection activity during the summer period and with tolerance to the salty soil area [28]. The carotenoid content in all the other examined organs was comparable as their values did not exhibit a significant difference. Concerning the anthocyanins, both stages of flowers (young blossom and ripe flowers) showed the highest amount of these secondary metabolites, which are related to the pigmentation of the flowers (Figure 1b), while they were not revealed in the leaves. Interesting results were obtained regarding the polyphenol content of the various examined organs. Both inflorescences showed the highest amount, found within the range of 17.15–18.42 mg GAE g^−1^ FW, while slightly lower content was observed in fresh wild leaves (4.62 mg). The obtained data on the polyphenolic content of wild plants were in agreement with those reported by Lee et al. [29].

The antioxidant activity, detected with two different assays (FRAP or DPPH), was therefore the highest in the flowers of *A. caerulescens*, followed by the leaves. The activity could be linked to the polyphenols, including the anthocyanins, since they work as scavengers of free radicals and as natural metal chelators [30], and to the carotenoids, which have achieved increasing interest in the last decades for their antioxidant properties [31]. On the other hand, the contribution of other antioxidant molecules can be included and further investigated.

Biochemical analyses of in vitro shoots were performed and referred to the different culture media. The chlorophyll content (Chl a, Chl b, and total chlorophyll) of shoots was higher in the MS-BA medium (581.04 µg g^−1^ FW) than in the basal MS medium (275.51 µg g^−1^ FW), and this effect was linked to the known influence of BA in chlorophyll production. Regarding the other pigments, carotenoids were higher in the MS-BA than in the MS medium, following the trend of the other photosynthetic pigments. Anthocyanins, belonging to the class of phenolic compounds, were more concentrated in the MS0 medium (1.03 mg ME g^−1^ FW) than in MS-BA (0.57 mg ME g^−1^ FW). In vitro shoots showed a reasonable polyphenol content (0.63–0.71 mg GAE g^−1^ FW), and the antioxidant activities (DPPH and FRAP assay) were higher in MS0 than in MS-BA (Table 2). Altogether, the contribution of cytokinin BA seemed to reduce the production of antioxidant compounds. A similar effect has already been observed in the shoot culture of *Scutellaria alpina* [32] and in the shoot culture of *Artemisia alba* [24], for which the high concentration of BA limited the amount of polyphenol compounds. The initial results obtained on *A. caerulescens* provide a basis for future investigations dealing with the optimization of the secondary metabolite production with the addition of plant growth regulators [24], or the addition of elicitors, or treatment with light, or precursor feedings, as already reported for the genus *Artemisia* [33]. 

### 2.3. Phytochemical Investigation

#### 2.3.1. Headspace Analysis

The complete composition of the headspaces (HSs) emitted by both the different parts of the wild plant (leaves, young inflorescences, and ripe inflorescences) and the in vitro shoots of *A. caerulescens* are reported in Table 3. A total of 35 compounds were identified, representing 97.3–100% of the whole volatile organic compounds (VOCs).

All the volatile emissions were characterized by the predominance of oxygenated monoterpenes, which resulted significantly higher in the HS of the in vitro culture (91.6%), followed by the ripe flowers (89.1%) > leaves (87.0%) > young inflorescences (70.2%). Among this class of compounds, thujones were the main constituent; in particular, α-thujone was revealed in remarkable percentages in all the samples, ranging from 45.4% in the young inflorescences and 83.2% in the in vitro shoots, whose HS was characterized by only ten compounds. β-thujone was detected in good percentages as well: it was higher in the samples which presented lower percentages of the α-isomer. The HS of young inflorescences exhibited the highest amount of this compound (11.2%), while the in vitro shoots had the lowest (7.6%). 

Monoterpene hydrocarbons were well-represented in the wild plant samples, whilst they were revealed in very low percentages (0.7%) in the volatilome of the in vitro culture. The young inflorescence HS presented the highest amount of this chemical class (27.2%), followed by the HSs of both the ripe inflorescences (9.8%) and leaves (9.3%). Sabinene was the main monoterpene hydrocarbon component detected in these samples as it accounted for 5.2% of content in the leaves and 16.1% and 6.5% in the young and ripe flowering tops, respectively. 

As well-evidenced in Table 3, the in vitro culture showed a less complex chemical composition of the volatile emission as compared to that of the different parts of the mother plant, probably due to a higher production of the oxygenated monoterpenes α- and β-thujones, representing the 83.2% and 7.6% of the whole aroma composition, respectively. The higher percentage of thujones in the in vitro shoots was probably attributable to a metabolism strongly directed to their production, as Dudareva et al. [34] reported a strong genetic regulation of the biosynthetic pathways involved in the volatile formation.

To the best of our knowledge, this is the first study on the chemical composition of the spontaneous volatile emission of *A. caerulescens,* although the compositions of the HSs of other *Artemisia* species have already been reported [35]. The main VOCs identified in the analysed samples were aligned with *Artemisia umbelliformis* subsp. *eriantha*, whose main compound was also α-thujone, although it is usually present in lower amounts than the β-isomer [36]. Conversely, the volatile profile of the analysed samples was very different from that of the *Artemisia argyi* H. Lév. studied by Li et al., who reported germacrene D (28.73%), α-pinene (16.44%), limonene (12.22%), cylcofenchene (6.46%), and α-phellandrene (4.06%) as the main components [37], and differing from that of *Artemisia spicigera* C. Koch and *Artemisia scoparia* Waldst. et Kit., as reported by Demirci et al., characterized mainly by camphor (37.5%) and β-pinene (20.8%), respectively [38]. Moreover, camphor, was also reported as a major component of *A. campestris* L. (31.78%), which was also rich in 1,8-cineole (23.11%) and α-thujone (16.82%) [39].

The data of the volatile composition of the plant headspaces (HSs) was subjected to multivariate statistical analysis with the use of the Hierarchical Cluster Analysis (HCA) and the Principal Component Analysis (PCA) methods. 

**Figure 2:**
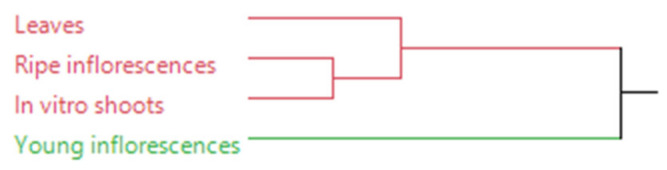
Dendrogram of the Hierarchical Cluster Analysis (HCA) performed on the complete chemical composition of the sample’s headspaces.

The dendrogram of the HCA, reported in Figure 2, showed two macro-clusters: the pink and green ones. The HS of the young inflorescences was clustered by itself in the green cluster, while the other samples were grouped together in the pink group. However, the HSs of ripe inflorescences and in vitro shoots were closer to each other than to the HS of the leaves.

**Figure 3:**
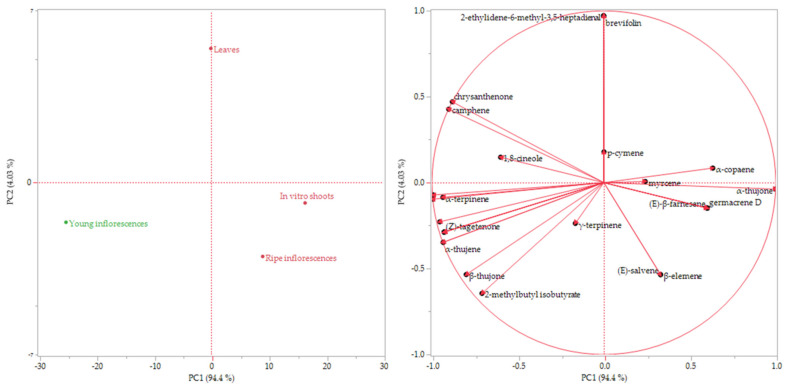
Score (**a**) and loading (**b**) plots of the principal component analysis (PCA) performed on the complete composition of the sample HSs.

The score and the loading plot of the PCA are reported in Figure 3a,b, respectively. The distribution of the samples was comparable to the partitioning of the HCA. The young inflorescence HS, which was clustered by itself in the dendrogram, was plotted in the bottom left quadrant in the score plot of the PCA (PC1 and PC2 < 0), probably for its considerable content of sabinene. The other three samples, which belong to the pink cluster, were plotted in the right quadrants (PC1 > 0); although the ripe inflorescences and the in vitro shoot HSs were located in the bottom quadrant (PC2 < 0), the HS of leaves was plotted on the partitioning line between the upper left and right quadrants, probably due to its higher content of 2-ethylidene-6-methyl-3,5-heptadienal.

Both graphs evidenced that the aroma composition of the in vitro shoots was not so different from the HSs of the different organs of the wild plant despite the fact that it was less complex because the thujones had the greatest impact on the plant fingerprint.

#### 2.3.2. Essential Oil Hydrodistillation

The EO yield was noticeable for all organs of the wild plants subjected to hydrodistillation as it ranged between 0.41% for the ripe inflorescence and 0.17% for the young inflorescences, passing through 0.35% for the leaves. This should be a positive aspect for the employment of the in vitro cultures to obtain this valuable extract. The hydrodistillation yield as well as the complete composition of the EOs obtained from the leaves, the young flowers, and the ripe ones of *A. caerulescens* are reported in Table 4. In total, 33 compounds were identified, representing 97.3–99.7% of the whole composition. 

The examined samples were characterized by the predominance of oxygenated monoterpenes, proving to be significantly more abundant in the EO obtained from the leaves (74.2%), than in both the young and ripe inflorescence EOs (65.4%). Among this chemical class, α-thujone was the most relevant compound, reaching 51.5% of the whole chemical composition in the EO of the leaves, 41.7% in the EO of ripe flowers, and 39.7% in the EO of young ones. β-thujone was also well-represented in all the samples, comprising a total of 9.3–12.0%. The chemical composition of the studied EOs was in accordance with Flamini et al. [35], who reported α- and β-thujone as the main compounds and the absence of camphor in the EO of *A. caerulescens* var. *palmata*. 

Tujones are volatile monoterpene ketones widely used as flavouring agents in the food industry. The accumulation of α- and β-thujone in the EOs is influenced by different external and internal factors, such as the plant genetic heritage, organ, and growth stage as well as weather and environmental conditions. Their biosynthesis, in fact, starts from geranyl-diphosphate (GPP) and neryl-diphosphate, through a four-step biosynthetic pathway, whose first produced monoterpene is sabinene. Despite sabinene, one of the most widespread monoterpene compound detected in the EOs, is the indirect precursor of thujones, their biosynthesis is restricted to only a few species as a consequence of plant genetic expression [44]. Unsurprisingly, since the metabolism was shifted to the production of thujones, the analysed samples showed low amounts of sabinene, ranging from 0.7 to 1.3% of the whole EO compositions. 

Today, thujones are the focus of debate concerning their effect on human health. Their presence in products intended for human consumption is regulated by the European Parliament and Council and the European Medicines Agency due to reported toxic effects related to their use. However, plants containing thujones have been widely employed as natural remedies in ethnobotanical applications. Recent investigations have cleared the mechanism of neurotoxicity in these chemicals as they are modulators of the GABA-gated chloride channels, but those studies have also evidenced several potential benefits such as their immune-modulatory and anti-carcinogenic properties as well as their antimicrobial effect. The effect of these chemicals seems to be strongly dose-dependent [44].

Moreover, the analysed samples were characterized by relevant amounts of chrysantenone, accounting for 6.6%, 8.1%, and 9.5% of the total amount in the EOs obtained from ripe inflorescences, young inflorescences, and leaves, respectively. The differences in the relative content of this component were noticeable between the sample headspace and the essential oil. In fact, chrysantenone was only detected in the HSs of leaves and young inflorescences, which amounted to 2.1% and 2.6% of the total amount, respectively. Despite the fact that this molecule has not ever been identified in *A. caerulescens* before, its presence was not surprising as it has been found to be one of the major components of the EO obtained from the aerial parts of *Artemisia herba-alba,* which showed high percentages of α- and β-thujone as well [45].

Phenylpropanoids were the second relevant class of compounds in all the analysed samples, resulting in higher representation in the EOs of both the young and the ripe inflorescences (28.8% and 27.9%, respectively) than in those obtained from the leaves (23.6%). The only detected compound belonging to this class was brevifolin, which has not been detected in *A. caerulescens* before but was revealed in good percentages in the EO obtained from *Artemisia turcomanica* Gand. [46]. 

## 3. Materials and Methods

### 3.1. Plant Materials

The spontaneous wild *Artemisia caerulescens* L. plants were harvested between June and September 2021 in a typical salt marsh called Lame, located in the San Rossore Estate, the heart of the Migliarino San Rossore Massaciuccoli Regional Park (Pisa, Italy). According to the most recent taxonomic revisions [47], the plants chosen for the study refer to *Artemisia caerulescens* subsp. *caerulescens* (=*Seriphidium caerulescens* (L.) Soják), previously cited under the no longer accepted name of *Artemisia caerulescens* L. var. *palmata* Lam. [43,44]. Reports on the species in the collection area are catalogued and georeferenced in the Wikiplantdbase #Toscana database (2015) [48]. Five plants in June (vegetative stage) and five plants during the blooming and the full-flowering phase (September) were used for the experiments. Leaves and flowers were directly used for the analysis of the volatile emission and the extraction of the essential oil. The aerial part of the plants (nodes) was also used for the in vitro propagation. The biochemical analyses were thus performed by the collection of fresh organs (leaves, flowers, in vitro shoots) and kept at −20 °C until use. In vitro shoots and the aerial organs of spontaneous plants (leaves, flowers) were used for biochemical and phytochemical analyses. 

### 3.2. In Vitro Shoot Proliferation

Aerial parts of spontaneous plants were divided into small nodal parts. Apical and nodal parts (1–1.5 cm) were used as explants and washed in Tween-20 for 30 min. The NaClO solution (20%) was used as a sterilization agent for 30 min, and the explants were further washed in sterilized water. These were subsequently placed in a culture medium composed of Murashige and Skoog salts and vitamins [19], 3% sucrose, and 0.8% agar, and the pH level was adjusted to 5.8 (called MS0). Other explants were placed in MS0 medium with the addition of 1, 2.4 μM 6-benzyl-aminopurine (BA, called MS-BA). The explants were maintained in a culture chamber at 23 ± 1 °C, with a 16/8 h light/dark cycle and 50 ± 5 μmol m^−2^ s^−1^ light irradiance. The subcultures were processed every 4 weeks.

### 3.3. Biochemical Analysis

#### 3.3.1. Pigment, Polyphenol, and Flavonoid Extraction and Determination

Ground/powdered fresh leaves of *A. caerulescens* (0.2 g each replicate) were incubated with 10 mL of 100% methanol for 24 h at 4 °C, and the absorbance was subsequently read at 665 nm, 652 nm, and 470 nm in a SHIMADZU UV-1800 spectrophotometer (Shimadzu^®^, Japan). Chlorophyll a, b, total chlorophyll, and carotenoid content were determined using the proper formulas reported by Lichtenthaler [49]. Biochemical determinations of total polyphenols (TP), total flavonoids (TF), and antioxidant activity were performed on extracts obtained by the homogenization of 0.2 g of the plant materials with 2 mL of 70% aqueous methanol, then kept for 30 min in ice, and centrifuged at 14,000× *g* for 20 min. The supernatants were used for the biochemical determinations. TPC was determined using a modified protocol of the Folin Ciocalteau method [50]. The analysis was carried out on 10 μL of the supernatant, in triplicates. The incubation was performed at 40 °C for 30 min, then the absorbance was spectrophotometrically determined at 765 nm. Total phenolic content (TP) was expressed as mg of GAE per g of DW (μg gallic acid equivalents per g FW). The total flavonoid content (TF) was determined as reported by Kim et al. [51] in 50 μL of the plant sample extracts. The absorbance was read at 415 nm and 510 nm, and the concentration was expressed as mg of (+)-catechin equivalents (CE) per g of FW.

#### 3.3.2. Antioxidant Activity 

The antioxidant activity of the fresh leaves, young inflorescences, and ripe inflorescences of the wild plants and the aerial part of the in vitro shoot of *A. caerulescens* was determined by using the DPPH (2,2-diphenyl-1-picrylhydrazyl radical) and FRAP (Ferric Reducing Antioxidant Power) scavenging methods [52]. The assays were performed in triplicates. 

Concerning the DPPH scavenging method, 20 μL aliquots of the methanolic extract were added to a 0.25 mM (*w*/*v*) DPPH methanol solution to reach a final volume of 1 mL. After 30 min of incubation at room temperature in the dark, the blenching of DPPH was measured at 517 nm. Trolox was used as control (2.5 mM), and the activity was expressed as µmol Trolox Eq g^−1^ FW. 

The scavenging activity of the sample by FRAP assay was performed using the Szôllôsi method [53]: aliquots of 20 μL of the sample were added to 900 μL of FRAP solution. After 4 min of incubation at room temperature, the absorbance was read at 593 nm. FeSO_4_ was used as standard, and the activity was expressed as mmol Fe^2+^ g^−1^ FW.

### 3.4. Phytochemical Investigation

#### 3.4.1. HS-SPME Analysis

The spontaneous volatile emission of the fresh roots, leaves, young inflorescences, and ripe inflorescences of the adult plant and the aerial part of the in vitro plant were analysed in triplicate by HS-SPME (Headspace Solid-Phase Microextraction). The samples (2 g each) were introduced into a 50 mL glass flask, subsequently covered with an aluminium foil, and left to equilibrate for 30 min at room temperature. Then, the headspaces were analysed using a Supelco PDMS fibre (100 μm) (Supelco analytical, Bellefonte, PA, USA), preconditioned according to the manufacturer’s instructions. The sampling of the headspaces were performed for 5 s for the leaves and the flowering tops of the adult plants, and for 15 s for the aerial part of the in vitro plant; the fibre was then withdrawn into the needle and immediately injected into the GC-MS apparatus.

#### 3.4.2. Essential Oil Hydrodistillation

The fresh leaves, the young inflorescences, and the ripe ones of A. caerulescens were hydrodistilled separately with a Clevenger-type apparatus (Tecnovetro Snc, Pisa, Italy) in order to obtain the essential oil. The hydrodistillation was performed in triplicates on 80 g of leaves, 40 g of young flowering tops, and 20 g of ripe inflorescences and was protracted for 2 h. Then, the collected EOs were diluted in 5–10% HPLC-grade n-hexane and injected into a GC-MS apparatus. 

#### 3.4.3. Gas Chromatography—Mass Spectrometry Analyses

The gas chromatography–electron impact mass spectrometry (GC–EIMS) analyses were performed with an Agilent 7890B gas chromatograph (Agilent Technologies Inc., Santa Clara, CA, USA) equipped with an Agilent HP-5MS capillary column (30 m × 0.25 mm; coating thickness 0.25 μm) and an Agilent 5977B single quadrupole mass detector. The analytical conditions for both the EOs and the SPME analyses were set as follows: oven temperature ramp from 60 to 240 °C at 3 °C/min; injector temperature, 220 °C; transfer line temperature, 240 °C; carrier gas helium, 1 mL/min. For the EO analyses, the injection volume was 1 μL, with a split ratio of 1:25. 

The acquisition parameters were the following: full scan; scan range: 30–300 *m*/*z*; scan time: 1.0 s. The identification of the constituents was based on a comparison of the retention times with those of pure samples, comparing their linear retention indices relative to the series of *n*-hydrocarbons. Computer matching was also used against commercial (NIST 14 and ADAMS 2007) and laboratory-developed mass spectra libraries, built up from pure substances and components of commercial essential oils of known composition and MS literature data [40,54,55,56,57,58].

### 3.5. Statistical Analysis

The analysis of variance (ANOVA) was performed using the JMP Pro 14.0.0 software package (SAS Institute, Cary, NC, USA). Concerning the phytochemical investigation, ANOVA analyses were carried out on the chemical classes of compounds for both the essential oil and the headspace analyses, and on the EO hydrodistillation yield. Concerning the biochemical analysis, ANOVA analyses were performed on chlorophyll A, chlorophyll B, total chlorophyll, total carotenoids, total polyphenols, total anthocyanins, radical scavenging assay (DPPH), and antioxidant activity (FRAP). Averages were separated by Tukey’s b post hoc test. *p* < 0.05 was used to assess the significance of differences between means. Multivariate statistical analyses were also performed with the JMP software package. Hierarchical cluster analysis (HCA) was performed using Ward’s method on unscaled data, with squared Euclidean distances as a measure of similarity. Principal component analysis (PCA) was performed on a 35 × 4 data covariance matrix (35 compounds × 4 samples = 140 data), selecting the two highest PCs, PC1 and PC2, obtained by the linear regression operated on the mean-centred, unscaled data, covering 94.40% and 4.03% of the variance, respectively, for a total variance of 98.03%. 

## 4. Conclusions

In the context of climatic changes, the micropropagation technique was useful in avoiding the germplasm depletion of species found in a protected area and in maintaining biodiversity. The preliminary results on the proliferation of in vitro shoots of *A. caerulescens,* a halophyte species still not fully characterized, showed the best results with the MS medium, added with 1 μM of BA. 

Biochemical analyses and antioxidant activity were investigated on both in vitro shoots and wild plants as well as the chemical composition of the spontaneous volatile emission, which, to the best of our knowledge, has never been studied before. Moreover, we also reported the chemical composition and the hydrodistillation yield of the essential oils obtained from the different organs of the plant. 

Total polyphenol content and antioxidant activity were noticeable in both the inflorescences, while leaves and in vitro shoots showed lower amounts. Due to the low amount of antioxidant compounds in the in vitro shoots, further studies are necessary to improve the production of secondary metabolites.

Concerning the phytochemical investigation, the oxygenated monoterpenes α- and β-thujone were the main compounds detected in both the analysed headspaces (HSs) and the essential oils (EOs). They are the representative compounds of the *Artemisia* genus, and their biological activity is widely used in folk medicine. The EO yield was noticeable in all the organs of the wild plant subjected to hydrodistillation. The EOs were also characterized by noticeable percentages of phenylpropanoids (23.6–28.8%), with brevifolin as the unique compound, which was not detected in the spontaneous volatile emissions of the same parts of the wild plant. The high percentage of phenylpropanoids identified in the EOs and the high EO yield represent an interesting starting point for future investigation into their biological activities that will promote their employment in other possible applications. 

## Figures and Tables

**Table 1:** In vitro culture of *A. caerulescens*. Single mother plants (M) were used as initial explants, and their contamination was observed (%). Effect of different culture media on shoot development (%). Proliferation and length of shoots (cm) after 4 weeks of cultivation. Data are presented as means ± SD (*n* = 10).

Mother Plant	Number of Explants	Contaminated Explants (%)	Developed Shoots (%)	Number of Shoots/Explant	Shoots Length (cm)
M1 (MS0)	18	32	88.9	1	2.67 ± 1.7
M2 (MS0)	10	0	100	1	3.5 ±1.6
M3 (MS0-BA)	20	90	0	0	0
M4(MS-BA 2 µM)	10	10	3	3	1.2 ± 0.4
M5(MS-BA 2 µM)	10	10	5	5	1.5 ± 0.5
MS0	10	0	100	2.1 ± 1.28 ^C^	3.18 ± 0.26 ^A^
MS-BA 1 µM	10	0	100	8.1 ± 1.63 ^A^	1.12 ± 0.14 ^C^
MS-BA 2 µM	10	0	100	5.4 ± 1.08 ^B^	1.5 ± 0.12 ^B^
MS-BA 4 µM	10	0	100	4.6 ± 1.27 ^B^	0.76 ± 0.11 ^D^

The superscript, uppercase letters (A–D) indicate statistically significant differences between the samples. The statistical significance of the relative abundances was determined by the Tukey’s post hoc test, with *p*  ≤ 0.05.

**Table 2:** Determination of metabolites in the different *A. caerulescens* organs grown in a wild area or in an in vitro culture. Data are presented as means ± SE (*n* = 5). Different letters indicate statistically significant differences determined by Tukey’s b post hoc test (*p* < 0.05). Abbreviations: nd.—not determined; GAE—gallic acid equivalents; CE—catechin equivalents; ME—malvin-chloride equivalents.

	Wild Plants			In Vitro Shoots	
	Leaves	Young Inflorescences (Blossom)	Ripe Inflorescences	Shoots (MS0)	Shoots (MS-BA 2 µM)
Chlorophyll a (Chl a, µg g^−1^ FW)	930.03 ± 12.19 ^A^	376.99 ± 0.23 ^BC^	231.31 ± 4.07 ^C^	208.9 ± 2.76 ^C^	448.25 ± 3.76 ^B^
Chlorophyll b (Chl b, µg g^−1^ FW)	182.27 ± 8.36 ^A^	114.78 ± 0.54 ^B^	68.71 ± 0.78 ^B^	66.6. ± 0.68 ^B^	132.79 ± 3.76 ^AB^
Total Chlorophyll (Tchl, 284.9 µg g^−1^ FW)	1112.3 ± 20.15 ^A^	491.76 ± 0.30 ^BC^	300.02 ± 4.51 ^C^	275.51 ± 3.44 ^C^	581.04 ± 14.54 ^B^
Total carotenoids (Tcar, µg g^−1^ FW)	294.9 ± 2.01 ^A^	134.39 ± 0.37 ^B^	91.47 ± 1.61 ^B^	60.2 ± 0.78 ^B^	124.11 ± 3.10 ^B^
Total Anthocyanins (TA, mg ME g^−1^ FW)	nd.	7.95 ± 0.51 ^AB^	20.47 ± 0.89 ^A^	1.03 ± 0.05 ^B^	0.57 ± 0.05 ^B^
Total Polyphenols (TP, mg GAE g^−1^ FW)	4.62 ± 0.13 ^B^	17.15 ± 0.12 ^A^	18.42 ± 0.24 ^A^	0.71 ± 0.01 ^B^	0.63 ± 0.05 ^B^
Radical scavenging DPPH-assay (µmol TEAC g^−1^ FW)	12.25 ± 0.55 ^B^	82.15 ± 4.80 ^A^	71.16 ± 2.94 ^A^	2.53 ± 0.10 ^B^	1.59 ± 0.16 ^B^
Antioxidant activity FRAP assay (mmol Fe^2+^ g^−1^ FW)	24.35 ± 0.86 ^B^	120.34 ± 3.64 ^A^	102.3 ± 1.53 ^AB^	4.91 ± 0.06 ^B^	4.30 ± 0.3 ^B^

The superscript, uppercase letters (A, B, C) indicate statistically significant differences between the samples. The statistical significance of the relative abundances was determined by the Tukey’s post hoc test, with *p* < 0.05.

**Table 3:** Complete chemical composition of the HSs emitted both by different parts of the wild plant (leaves, young inflorescences, and ripe inflorescences) and the 4-week-old in vitro shoots of *A. caerulescens* (*n* = 3. ± SD).

Compounds	l.r.i. ^1^	l.r.i. ^2^	Class	Relative Abundance ± Standar Deviation (%)			
				Leaves	Young Inflorescences (Blossom)	Ripe Inflorescences	In Vitro Shoots
ethyl 2-methylbutyrate	850	850	nt	- ^3^	0.1 ± 0.00	-	-
*(E)-*salvene	867	867	nt	-	-	0.4 ± 0.15	-
ethyl isovalerate	859	858	nt	-	1.9 ± 0.22	-	-
tricyclene	922	927	mh	-	0.3 ± 0.00	-	
α-thujene	926	1102	mh	0.1 ± 0.07	0.6 ± 0.11	0.2 ± 0.06	-
α-pinene	933	939	mh	0.9 ± 0.18	3.2 ± 0.05	0.3 ± 0.09	-
camphene	948	954	mh	0.6 ± 0.09	0.8 ± 0.04	-	-
sabinene	973	975	mh	5.2 ± 0.82	16.1 ± 1.06	6.5 ± 2.04	-
β-pinene	977	979	mh	0.1 ± 0.02	0.6 ± 0.03	0.2 ± 0.08	-
myrcene	991	991	mh	0.2 ± 0.06	0.3 ± 0.02	-	0.7 ± 0.38
2-methylbutyl isobutyrate	1016	1017	nt	-	0.3 ± 0.03	0.2 ± 0.02	-
α-terpinene	1017	1017	mh	0.1 ± 0.02	0.2 ± 0.01	0.1 ± 0.01	-
*p*-cymene	1025	1025	mh	1.4 ± 0.16	0.6 ± 0.03	1.7 ± 0.25	-
limonene	1029	1029	mh	0.3 ± 0.05	0.9 ± 0.17	0.2 ± 0.05	-
1,8-cineole	1031	1031	om	1.2 ± 0.04	1.2 ± 0.06	1.2 ± 0.14	0.8 ± 0.22
γ-terpinene	1058	1060	mh	0.3 ± 0.02	0.3 ± 0.00	0.6 ± 0.10	-
α-thujone	1107	1102	om	67.9 ± 1.56	45.4 ± 0.04	78.1 ± 2.56	83.2 ± 1.41
β-thujone	1117	1114	om	8.0 ± 0.64	11.2 ± 1.80	9.7 ± 0.28	7.6 ± 0.16
chrysanthenone	1126	1128	om	2.1 ± 0.43	2.6 ± 0.54	-	-
*trans*-pinocarveol	1139	1139	om	0.2 ± 0.03	-	-	-
*(Z)-*tagetenone	1231	1229	om	-	0.6 ± 0.02	-	-
*(E)-*tagetenone	1240	1238	om	0.3 ± 0.02	-	-	-
*iso*piperitenone	1271	1272 *	om	0.7 ± 0.02	-	-	-
perilla aldehyde	1273	1272	om	-	0.5 ± 0.30	-	-
*(E,E)*-2,4-decadienal	1316	1317	nt	0.2 ± 0.03	-	-	-
cyclosativene	1367	1371	sh	-	-	-	1.2 ± 0.26
α-copaene	1376	1377	sh	0.4 ± 0.06	-	-	1.7 ± 0.10
β-elemene	1392	1391	sh	-	-	0.6 ± 0.11	-
2-ethylidene-6-methyl-3,5-heptadienal	1395	1395	om	6.4 ± 1.01	-	-	-
cyperene	1399	1399	sh				2.0 ± 0.68
*(E)-*β-farnesene	1458	1457	sh				1.3 ± 0.17
germacrene D	1481	1485	sh				0.8 ± 0.20
β-selinene	1486	1490	sh	0.3 ± 0.02	-	-	-
T-cadinol	1641	1640	os				0.7 ± 0.10
brevifolin	1669	1675 *	pp	0.2 ± 0.03	-	-	-
Total identified (%)				97.3 ± 0.06	100.0 ± 0.01	100.0 ± 0.01	100.0 ± 0.01
Chemical Classes				Leaves	Young Inflorescences (Blossom)	Ripe Inflorescences	In Vitro Shoots
Monoterpene hydrocarbons (mh)				9.3 ± 0.95 ^B^	27.2 ± 0.13 ^A^	9.8 ± 2.66 ^B^	0.7 ± 0.38 ^C^
Oxygenated monoterpenes (om)				87.0 ± 0.97 ^B^	70.2 ± 0.34 ^C^	89.1 ± 2.72 ^AB^	91.6 ± 1.35 ^A^
Sesquiterpene hydrocarbons (sh)				0.6 ± 0.08 ^B^	- ^B^	0.6 ± 0.11 ^B^	7.0 ± 0.88 ^A^
Oxygenated sesquiterpenes (os)				- ^B^	- ^B^	- ^B^	0.7 ± 0.10 ^A^
Phenylpropanoids (pp)				0.2 ± 0.03	-	-	-
Other non-terpene derivatives (nt)				0.2 ± 0.03 ^C^	2.6 ± 0.20 ^A^	0.5 ± 0.17 ^B^	- ^C^

^1^ Linear retention index experimentally determined on an HP 5-MS capillary column; ^2^ Linear retention index reported in the literature by Adams 2007 [40], NIST 14 [41], and NIST Chemistry WebBook [42]; * linear retention time in PubChem [43]. ^3^ Not detected. For the chemical classes and for the superscript, uppercase letters (A, B, C) indicate statistically significant differences between the samples. The statistical significance of the relative abundances was determined by the Tukey’s post hoc test, with *p*  ≤ 0.05.

**Table 4:** Complete chemical composition and hydrodistillation yield of the essential oils obtained from fresh leaves and young and ripe inflorescence samples of *A. caerulescens* (*n* = 3; ±SD).

Compounds	l.r.i. ^1^	l.r.i. ^2^	Class	Relative Abundance (%) ± SD		
				Leaves	Young Inflorescences (Blossom)	Ripe Inflorescences
ethyl isovalerate	859	858	nt	- ^3^	0.1 ± 0.03	-
α-pinene	933	939	mh	-	0.2 ± 0.03	0.1 ± 0.00
sabinene	973	975	mh	0.7 ± 0.18	1.3 ± 0.01	1.2 ± 0.02
*p*-cymene	1025	1025	mh	0.2 ± 0.04	-	0.3 ± 0.03
1,8-cineole	1031	1031	om	0.3 ± 0.01	0.3 ± 0.02	0.3 ± 0.01
γ-terpinene	1058	1060	mh	-	0.1 ± 0.01	0.1 ± 0.01
*cis*-sabinene hydrate	1066	1070	om	0.3 ± 0.04	0.3 ± 0.02	0.4 ± 0.05
*trans*-sabinene hydrate	1098	1098	om	0.1 ± 0.03	0.2 ± 0.01	0.5 ± 0.04
α-thujone	1107	1102	om	51.5 ± 0.03	39.7 ± 1.81	41.7 ± 0.78
filifolone	1108	1109	om	1.6 ± 0.54	-	-
β-thujone	1117	1114	om	9.3 ± 1.45	12.0 ± 0.07	9.9 ± 0.27
dehydrosabinaketone	1119	1121	om	-	0.2 ± 0.05	-
chrysanthenone	1126	1128 *	om	9.5 ± 1.24	8.1 ± 1.23	6.6 ± 0.02
*trans*-pinocarveol	1139	1139	om	0.5 ± 0.05	0.4 ± 0.03	1.2 ± 0.07
pinocarvone	1163	1165	om	0.3 ± 0.04	0.2 ± 0.02	1.0 ± 0.01
4-terpineol	1177	1177	om	0.4 ± 0.08	0.4 ± 0.00	0.3 ± 0.00
myrtenal	1194	1196	om	0.1 ± 0.02	0.1 ± 0.00	-
piperitone	1254	1253	om	0.2 ± 0.05	-	-
*cis*-chrysanthenyl acetate	1262	1265	om	-	0.4 ± 0.03	0.8 ± 0.01
*iso*piperitenone	1271	1272 *	om	0.2 ± 0.03	0.1 ± 0.02	-
*trans*-sabinyl acetate	1294	1291	om	-	3.1 ± 0.41	2.0 ± 0.09
β-caryophyllene	1419	1419	sh	-	-	0.3 ± 0.02
germacrene D	1481	1485	sh	0.5 ± 0.27	0.8 ± 0.15	-
β-selinene	1486	1490	sh	-	1.0 ± 0.07	1.7 ± 0.14
phenylethyl 3-methylbutyrate	1491	1490	nt	0.3 ± 0.20	-	-
α-cadinol	1654	1654	os	-	0.1 ± 0.02	-
brevifolin	1669	1675	pp	23.6 ± 1.21	27.9 ± 2.21	28.8 ± 1.07
mustakone	1687	1676	os	-	0.3 ± 0.01	-
Eudesma-4(15),7-dien-1β -ol	1688	1688	os	-	0.3 ± 0.04	-
β-nootkatol	1712	1712	os	-	0.3 ± 0.01	-
*(Z)-*lanceol	1762	1761	os	-	1.0 ± 0.14	-
methyl *iso*costate	1792	1791	os	-	0.7 ± 0.21	-
kaurene	2048	2043	dh	-	0.2 ± 0.01	-
Chemical Classes				Leaves	Young Inflorescences (Blossom)	Ripe Inflorescences
Monoterpene hydrocarbons (mh)				0.9 ± 0.22 ^B^	1.7 ± 0.03 ^A^	1.8 ± 0.05 ^A^
Oxygenated monoterpenes (om)				74.2 ± 2.01 ^A^	65.4 ± 2.74 ^B^	64.7 ± 1.02 ^B^
Sesquiterpene hydrocarbons (sh)				0.5 ± 0.27 ^B^	1.7 ± 0.22 ^A^	2.0 ± 0.16 ^A^
Oxygenated sesquiterpenes (os)				-	2.7 ± 1.59	-
Phenylpropanoids (pp)				23.6 ± 1.21 ^B^	27.9 ± 2.21 ^A^	28.8 ± 1.07 ^A^
Other non-terpene derivatives (nt)				0.3 ± 0.20	0.1 ± 0.03	-
Total identified (%)				99.5 ± 0.12	99.7 ± 0.00	97.3 ± 0.15
OE hydrodistillation yield (% *w*/*w*)				0.35 ± 0.01 ^B^	0.17 ± 0.01 ^C^	0.41 ± 0.01 ^A^

^1^ Linear retention index experimentally determined on an HP 5-MS capillary column; ^2^ Linear retention index reported in the literature by Adams 2007 [40], NIST 14 [41], and NIST Chemistry WebBook [42]; * linear retention time in PubChem [43]. ^3^ Not detected. For the chemical classes and the EO hydrodistillation yield, the superscript uppercase letters (A, B, C) indicate statistically significant differences between the samples. The statistical significance of the relative abundances was determined by the Tukey’s post hoc test, with *p*  ≤ 0.05.

## Data Availability

The data used in this work are new and original, and they are fully reported in the present manuscript.
